# Forensic age estimation in living adolescents with CT imaging of the clavicula—impact of low-dose scanning on readers’ confidence

**DOI:** 10.1007/s00330-020-07079-y

**Published:** 2020-07-28

**Authors:** Sebastian Gassenmaier, Juergen F. Schaefer, Konstantin Nikolaou, Michael Esser, Ilias Tsiflikas

**Affiliations:** Department of Diagnostic and Interventional Radiology, University Hospital Tuebingen, Hoppe-Seyler-Straße 3, 72076 Tuebingen, Germany

**Keywords:** Forensic medicine, Multidetector computed tomography, Age determination by skeleton, Radiation dosage, Sternoclavicular joint

## Abstract

**Objectives:**

Computed tomography (CT) imaging of the clavicula displays the reference standard for forensic bone age diagnostics in adolescents and young adults. Consequently, highest efforts on radiation reduction are warranted. Therefore, the aim of this study was to investigate the feasibility of low-dose (LD) CT imaging of the clavicula for age estimation in living adolescents.

**Methods:**

A total of 207 non-contrast chest CT of 144 patients born between 1988 and 2012, performed in 2018 due to various clinical indications, were included in this retrospective study. The mean patient age was 16.9 ± 6.6 years. Patients were divided into a LD (*n* = 146) and standard-dose (SD; *n* = 61) group. Image quality, confidence levels, and ossification stages (using the 5-stage classification including the subgroups 2a–3c) were assessed by two radiologists independently. Radiation dose was determined via dosimetry software.

**Results:**

Dose simulation with *z*-axis reduction to depict the clavicula only resulted in a median exposure of 0.1 mSv (IQR: 0.0) in LD compared with 0.9 mSv (IQR: 0.6) in SD (*p* < 0.001). The median image quality was rated by both readers significantly worse in LD compared with SD on a Likert scale ranging from 1 to 4 with a median of 3 (IQR: 1) versus 4 (IQR: 0; *p* < 0.001 for both readers). There was an almost perfect agreement for the ossification stages between both readers with a Cohen’s kappa of 0.83 (*p* < 0.001). Median confidence levels of both readers were not significantly different between LD and SD in the decisive subgroups 2a–3c.

**Conclusions:**

Low-dose CT imaging of the clavicula for age estimation in living adolescents is possible without loss of readers’ confidence.

**Key Points:**

*• Radiological bone age diagnostics in young delinquents with unknown exact chronological age is important as the judicial systems differentiate between youths and adults.*

*• Low-dose computed tomography scanning of the medial clavicular joint for forensic age estimation is feasible in living adolescents without loss of readers’ confidence.*

*• Sufficient image quality of the medial clavicular joint for forensic bone age diagnostics in living adolescents is achievable using a median dose of 0.1 mSv.*

## Introduction

X-ray imaging of the left hand for bone age estimation in living subjects using the atlas of Greulich and Pyle displays a standard procedure in radiology [1]. However, this method is connected with a certain margin of error in adolescents after fully completed skeletal development of the left hand [1–3]. Therefore, other methods involving X-ray or magnetic resonance imaging (MRI) of the wrist, pelvis, and knee have been introduced by several authors for more reliable age estimation [4–7]. The current radiological reference standard for forensic age estimation in living adolescents is displayed by computed tomography (CT) imaging of the medial clavicular epiphysis [3, 8–10]. This method was firstly introduced by Kreitner et al in 1997 using a four-stage classification for age estimation [9]. In later studies, a fifth stage was appended for more precise age estimation [11]. Although in some investigations conventional radiography of the medial clavicular epiphysis was applied, several studies pointed out that the data provided by CT imaging is superior [12–14]. Due to the technical development of CT scanners including thin slice acquisition, the five-stage classification was further edited by Kellinghaus et al with the introduction of more detailed subgroups 2a–c and 3a–c [15, 16].

Forensic age estimation in living adolescents is carried out for medicolegal issues only, mostly due to severe criminal actions and the resulting juridical implications. Therefore, greatest efforts are required to reduce radiation dose as the imaging does not contribute to the diagnosis of a disease in these young delinquents. The ALARA (as low as reasonably achievable) principle should always be observed.

Therefore, the aim of this study was to investigate the feasibility of low-dose (LD) CT imaging of the clavicula for forensic age determination of living adolescents and its impact on image quality as well as on the confidence level of the reader.

## Materials and methods

### Study characteristics

**Fig. 1:**
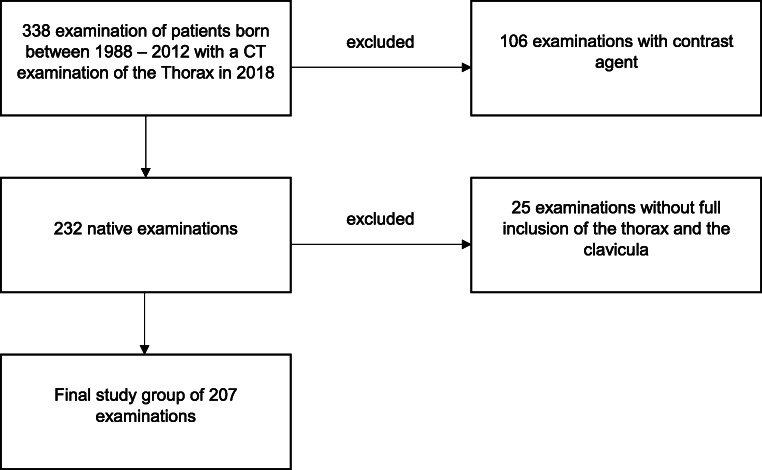
Flowchart of this study

This monocentric, retrospective study was approved by the institutional review board. All CT examinations of the chest which were performed in our institution in 2018 were searched via the radiology information system for the subgroup of patients born between 1988 and 2012 (*n* = 338). Patients born prior to 1988 or after 2012 were excluded due to expected complete skeletal maturation or fully immature stage of ossification. In a next step, all contrast-enhanced examinations were excluded to achieve homogeneity of the study group, resulting in 232 non-contrast examinations. Imaging studies which did not depict the complete thorax including the medial sternoclavicular joint were also removed from the cohort. This resulted in the final study group of 207 examinations in 144 patients (Fig. 1).

### Imaging protocols and dose evaluation

The CT examinations were performed using three different scanners (Siemens SOMATOM Force, Siemens SOMATOM Definition Flash, Siemens SOMATOM Definition AS+; all Siemens Healthineers). Imaging was conducted in supine position and in cranio-caudal scanning direction with a collimation of 0.6 mm after scout acquisition. Two different protocols were used for LD imaging: the first protocol consisted of a tube voltage of 100 kV with tin filtration and a reference tube current of 96 mAs using automated adaptation of tube current (CARE Dose; Siemens Healthineers); the second protocol involved automated adaptation of tube voltage and tube current (CARE kV and CARE Dose; Siemens Healthineers), using a reference tube voltage of 100 kV without tin filtration and a reference tube current of 10 mAs. For standard-dose (SD) imaging, a tube voltage of 90–120 kV without tin filtration and a reference tube current of 55–123 mAs depending on the scanner were applied. One-millimeter slice thickness and 1-mm increment applying a high-frequency (bone) kernel in transversal and coronal planes were used for image reconstruction. Iterative algorithms (SAFIRE or ADMIRE; Siemens Healthineers) were used as in clinical routine. Patients were divided into two different groups for comparison, according to the imaging protocol, which resulted in 146 LD vs. 61 SD examinations.

Dose length product (DLP), computed tomography dose index (CTDI_vol_), and effective dose (ED) were evaluated using a commercially available dosimetry and tracking software (Radimetrics; Bayer Healthcare). Effective dose was calculated according to the International Commission on Radiological Protection 103 (ICRP 103). Additionally, a dose simulation with reduced *z*-axis scanning which involved the whole clavicula only was performed via this software.

### Image evaluation

Image evaluation was performed independently by two radiologists with 1 year and 13 years of experience on a dedicated workstation (GE Centricity PACS RA 1000; General Electric Healthcare), respectively. Images were evaluated in axial and coronal planes using a slice thickness of 1 mm. The presence of artifacts was noted using a nominal scale. Image quality was evaluated according to a Likert scale ranging from 1 (non-diagnostic) to 4 (excellent). The previously published ossification stages by Schmeling et al (stages 1–5) and the further subclassification of Kellinghaus et al (2a, b, c and 3a, b, c) were used for age estimation [11; 15]:

- Stage 1: epiphyseal ossification center not ossified
- Stage 2a: less than one-third of the ossification center ossified
- Stage 2b: less than two-thirds but more than one-third of the ossification center ossified
- Stage 2c: more than two-thirds of the ossification center ossified
- Stage 3a: less than one-third of the epiphyseal cartilage ossified
- Stage 3b: less than two-thirds but more than one-third of the epiphyseal cartilage ossified
- Stage 3c: more than two-thirds of the epiphyseal cartilage ossified
- Stage 4: full ossification with visible scar
- Stage 5: full ossification without visible scar

In the case of two different stages between both medial clavicular joints within one patient, the higher stage was chosen. Additionally, each reader stated the confidence level of the age estimation on a Likert scale ranging from 1 (low) to 4 (very high). A subset of 32 pairs of examinations which were scanned twice within 100 days was used for intra-reader agreement.

### Statistical analysis

Proprietary statistical software was used for evaluation (IBM SPSS Statistics, version 23; JMP 14, SAS Institute). Normal distribution of the variables was assessed with the Shapiro-Wilk test. The Student’s *t* test and the Wilcoxon signed rank-sum test were used for independent parametric and non-parametric data. In case of paired data, the dependent *t* test and the paired Wilcoxon signed rank test were applied. For unpaired and paired binominal data, the chi-square test and McNemar test were used, respectively. Spearman’s correlation was applied for ordinal scaled units between both radiologists. Cohen’s kappa was applied for inter- and intra-reader agreement. The significance level alpha was set at 0.05.

## Results

### Patients’ characteristics

A total of 207 examinations were evaluated. The mean patient age was 16.9 ± 6.6 years (range, 5.4–29.8 years). A total of 112 examinations were performed in male patients. Further characteristics are displayed in Table 1.

**Table 1:** Characteristics of the study group

Characteristics	Values
Examinations Patients	*n* = 207 (112 in male patients) *n* = 144
Age at examination date	
Mean age ± std.	16.9 ± 6.6 years
Range	5.4–29.8 years
Low-dose group (LD)	
Number of examinations	*n* = 146
Mean age ± std.	13.7 ± 4.7 years
Range	5.4–28.8 years
Standard-dose group (SD)	
Number of examinations	*n* = 61
Mean age ± std.	24.5 ± 3.4 years
Range	15.3–29.8 years
Subset 2a–3c	
Number of examinations	*n* = 65 (LD: *n* = 45; SD: *n* = 20)
Mean age ± std.	LD: 17.1 ± 2.1 years; SD: 21.4 ± 3.0 years
Range	LD: 13.9–21.5 years; SD: 15.3–29.8 years

### Radiation dose exposure

Median DLP was significantly lower in LD scans with 8.3 mGy*cm (interquartile range (IQR): 7.0) vs. 226.8 mGy*cm (IQR: 68.5) in SD scans (*p* < 0.001). Accordingly, median ED was also lower in LD scans with 0.2 mSv (IQR: 0.1) vs. 4.6 mSv (IQR: 2.2) in SD examinations (*p* < 0.001). Results of dose simulation with *z*-axis reduction to the range of the clavicula displayed a significantly lower radiation exposure with a median simulated ED of 0.1 mSv (IQR: 0.0) in the LD group vs. 0.9 mSv (IQR: 0.6) in the SD group (*p* < 0.001) (Table 2).

**Table 2:** Comparison of radiation dose between low-dose and standard-dose imaging

	Low-dose imaging	Standard-dose imaging	*p* value
DLP (mGy*cm)	8.3 (IQR: 7.0)	226.8 (IQR: 68.5)	< 0.001
CTDI_vol_ (mGy)	0.3 (IQR: 0.2)	6.7 (IQR: 1.8)	< 0.001
Effective dose (mSv)	0.2 (IQR: 0.1)	4.6 (2.2)	< 0.001
Simulated effective dose^1^ (mSv)	0.1 (IQR: 0.0)	0.9 (IQR: 0.6)	< 0.001

^1^Analysis of 55 low-dose and 50 standard-dose cases

*DLP* dose length product, *CTDI*_*vol*_ computed tomography dose index

### Image quality assessment

**Fig. 2:**
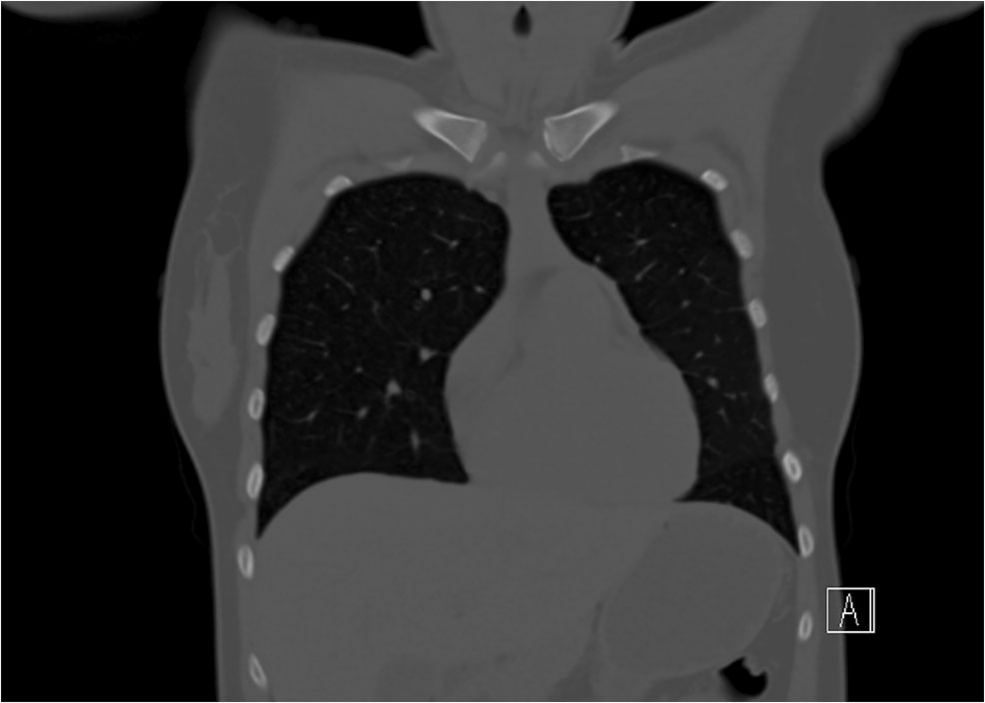
Coronal view of a chest CT using a standard-dose imaging protocol (120 kV/reference 100 mAs; effective dose 6.1 mSv). The ossification stage was classified as 2c

**Fig. 3:**
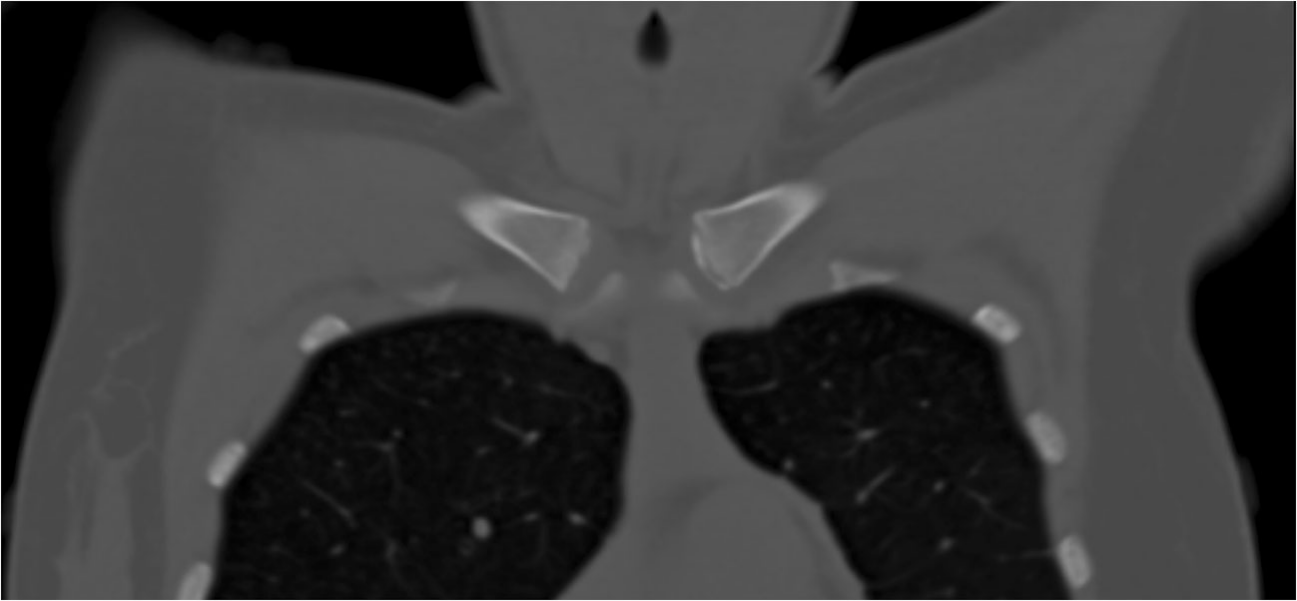
Magnification of the medial clavicular joint of Fig. 2 (standard-dose)

**Fig. 4:**
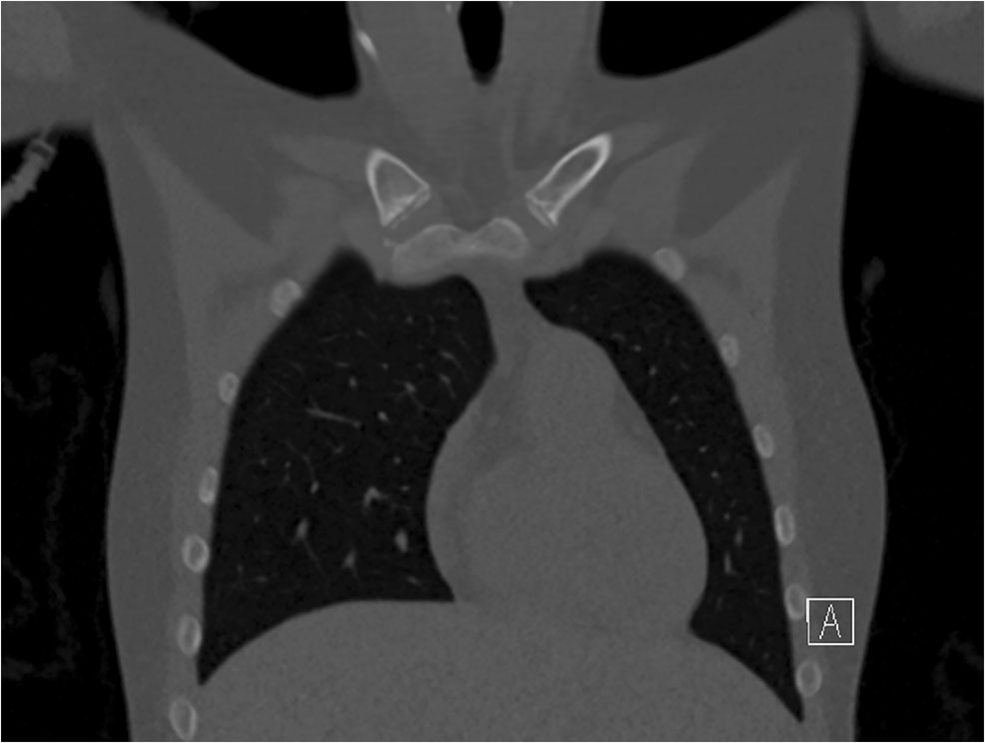
Coronal view of a chest CT using a low-dose imaging protocol (Sn100/reference 96 mAs; effective dose: 0.4 mSv). The ossification stage was classified as 2c

**Fig. 5:**
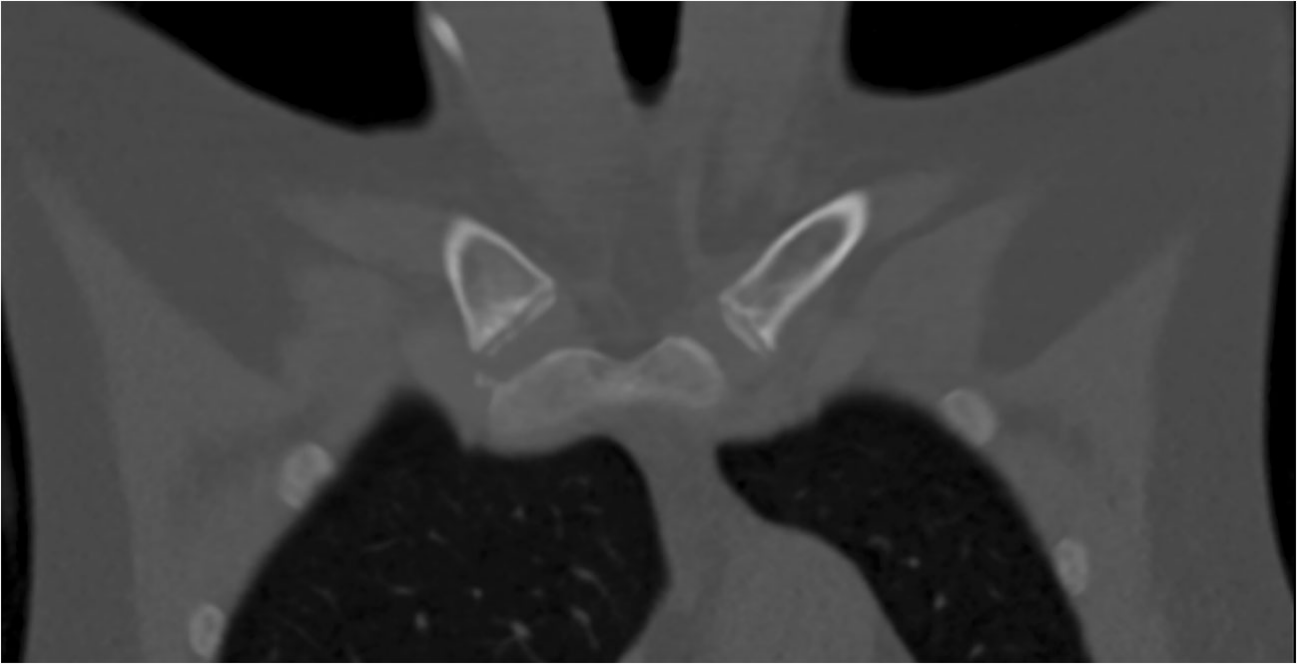
Magnification of the medial clavicular joint of Fig. 4 (low-dose)

Median image quality in SD imaging was 4 (IQR: 0). The image quality in LD imaging was rated significantly lower, with a median of 3 (IQR: 1) by both readers (*p* < 0.001 for both readers). There was almost perfect agreement between both readers concerning image quality assessment, with a value of 0.83 (*p* < 0.001; Table 3). There was no significant difference regarding the presence of artifacts (*p* = 0.517). Both readers found one motion artifact in the same LD examination while no artifact was found in SD imaging. Figures 2, 3, 4, and 5 display an example for LD and SD imaging.

**Table 3:** Correlation and inter-reader variability analysis

	Spearman’s correlation		Inter-reader variability	
	Correlation	*p* value	Kappa	*p* value
Image quality (*n* = 207) Low-dose (*n* = 146) Standard-dose (*n* = 61)	0.913 0.809 1	< 0.001 < 0.001	0.831 0.728 1	< 0.001 < 0.001
Ossification stages (*n* = 207) Low-dose (*n* = 146) Standard-dose (*n* = 61)	0.985 0.975 0.913	< 0.001 < 0.001 < 0.001	0.934 0.950 0.855	< 0.001 < 0.001 < 0.001
Subset 2a–3c (*n* = 65) Low-dose (*n* = 45) Standard-dose (*n* = 20)	0.986 0.970 0.986	< 0.001 < 0.001 < 0.001	0.925 0.914 0.938	< 0.001 < 0.001 < 0.001

### Ossification stages and confidence levels of the whole study group

There was a significant age difference between the LD group with 13.7 ± 4.7 years vs. 24.5 ± 3.4 years in the SD group (*p* < 0.001). The most often ossification stage in LD examinations was stage 1 in both readers (95 vs. 96 cases) while the SD group exhibited most often stage 4 in both readers (28 vs. 32 cases). There was no significant difference between both readers regarding the ossification stages (*p* = 0.405). Spearman’s correlation between both radiologists was 0.985 (*p* < 0.001). Overall, there was almost perfect agreement between both readers with a kappa of 0.934 (*p* < 0.001). The analysis of the LD group only resulted in a Spearman’s correlation of 0.975 and in an inter-reader agreement of 0.950 (both *p* < 0.001). In SD imaging, Spearman’s correlation was 0.913 and the inter-reader agreement analysis showed a kappa of 0.855 (both *p* < 0.001; Table 3).

Median confidence levels of both readers were significantly higher in LD imaging with 4 (IQR: 0) vs.4 (IQR: 1) in SD imaging (*p* = 0.035 for reader 1, *p* = 0.021 for reader 2; Table 4).

**Table 4:** Confidence analysis depending on the imaging protocol

	Low-dose imaging	Standard-dose imaging	*p* value
Confidence level; median (IQR)			
Reader 1	4 (0)	4 (1)	0.035
Reader 2	4 (0)	4 (1)	0.021
Subset 2a–3c analysis; median (IQR)			
Reader 1	4 (1)	3.5 (1)	0.186
Reader 2	3 (1)	3 (2)	0.074

*IQR* interquartile range

### Ossification stages and confidence levels of the subset 2a–3c

A further analysis of a subset of 65 examinations with ossification stages 2a–3c was performed as these stages are the most difficult ones to differentiate. The mean patient age in this subset was 17.7 ± 2.1 years in LD imaging (45 cases) and 21.4 ± 3.0 years in SD imaging (20 cases). There was no significant difference between both radiologists regarding the ossification stages 2a–3c (*p* = 0.317). Spearman’s correlation of the ossification stages between both readers was 0.986 (*p* < 0.001). Although a slight decrease in the inter-reader agreement could be observed, it was still almost perfect with 0.925 (*p* < 0.001; Table 3). Median confidence level of reader 1 was 4 (IQR: 1) in LD imaging and 3.5 (IQR: 1) in SD imaging (*p* = 0.186). Median confidence level of reader 2 was 3 (IQR: 1) in LD imaging and 3 (IQR: 2) in SD imaging (*p* = 0.074; Table 4).

### Intra-reader variability assessment of the ossification stages

A further subset of 32 pairs of examinations (25 pairs of LD imaging) which were performed within 100 days were used for intra-reader variability assessment of the ossification stages. Intra-reader agreement for both readers was 0.765 (*p* < 0.001).

## Discussion

This study could show that LD CT imaging of the clavicula for forensic age determination in living subjects is feasible without loss of readers’ confidence. Although the image quality was rated worse in the LD group, this did not affect the confidence levels of the readers regarding age estimation.

This is to our knowledge the first study investigating the feasibility of LD scans of the clavicula for forensic age estimation. As forensic imaging is carried out for medicolegal issues only, utmost efforts for radiation exposure reduction are necessary. It was shown previously that medical imaging using X-rays can cause DNA damage [17]. This is especially relevant as children and adolescents are more sensitive to ionizing radiation due to biological factors [18]. Additionally, adolescents exposed to radiation are more likely to develop cancer than adults due to their higher life expectancy and higher rates of cell division [19–21]. Large epidemiological studies have shown previously that patients who were exposed to CT examinations during their childhood exhibit higher cancer rates [22–24]. However, it is difficult to further specify the exact attributable risk of X-ray examinations due to the complexity of population-based studies and the inherently associated confounders [22, 25, 26].

These above mentioned issues strengthen the importance of radiation reduction. In our study, two different LD imaging approaches were followed which resulted in a median radiation exposure in LD imaging of the clavicula of 0.1 mSv. This corresponds to an equivalent of approximately 2 weeks of natural background radiation [18]. Therefore, the slightly higher radiation dose compared with conventional radiography can be considered tolerable due to the higher robustness and reliability of CT data [13, 14]. Although it was previously shown that also MRI of the medial clavicular epiphysis can be used for age estimation, the exact role and reliability of MRI remain still unclear in this area [27].

The high inter-reader agreement between both readers with different levels of experience in our study indicates that forensic age determination via CT of the clavicula seems to be a manageable challenge. Therefore, the application of LD CT imaging of the clavicula should not be reserved for highly specialized centers, only. Similar to the analysis of the skeletal bones of the left hand using the common radiographic atlas of skeletal development by Greulich and Pyle, bone age classification via CT should become a standard procedure [1].

It is striking that the analysis of the whole study group revealed higher confidence levels for the LD group compared with SD imaging. This is probably due to the high number of patients with the easily identifiably ossification stage 1 in the LD group. Therefore, a further subset analysis of the stages 2a–3c was performed which showed no significant difference of confidence between LD and SD imaging. This is especially important as the stages 2a–3c are the most difficult to differentiate. Additionally, the exact classification of adolescents within this range of age plays a vital role for further implications resulting from the categorization as juvenile or adult delinquent.

## Limitations

Our study was a monocentric, retrospective study using mostly modern dual-source generation scanners equipped with all technical benefits such as tin filtration and iterative reconstruction algorithms. A further limitation that merits consideration is the different group size of patients in LD and SD imaging. Additionally, the patients in the SD group were slightly older, as LD imaging protocols were preferably applied in very young patients. Most patients suffered from a malignant primary disease with a possible effect on the bone age. However, the aim of this study was not to compare bone age with chronological age but to analyze the feasibility and impact of a LD imaging protocol. Additionally, CT imaging is no primary diagnostic tool in this young patient cohort. Consequently, almost no CT studies were available from patients without severe primary disease, though further prospective studies are necessary to confirm these initial results on a broader spectrum of patients and different scanner architectures.

## Conclusions

The results of this study indicate that LD CT imaging of the medial clavicular joint for age estimation is possible without loss of readers’ confidence. By restricting the scanning area to the clavicula only, the radiation exposure can be reduced to an equivalent of approximately 2 weeks of natural background radiation.

## Abbreviations

CT: Computed tomography
CTDIvol: Computed tomography dose index
DLP: Dose length product
ED: Effective dose
ICRP 103: International Commission on Radiological Protection 103
IQR: Interquartile range
LD: Low-dose
MRI: Magnetic resonance imaging
SD: Standard-dose
