# Correction to: Forensic age estimation in living adolescents with CT imaging of the clavicula—impact of low-dose scanning on readers’ confidence

**DOI:** 10.1007/s00330-021-08111-5

**Published:** 2021-06-22

**Authors:** Sebastian Gassenmaier, Juergen F. Schaefer, Konstantin Nikolaou, Michael Esser, Ilias Tsiflikas

**Affiliations:** Department of Diagnostic and Interventional Radiology, University Hospital Tuebingen, Hoppe-Seyler-Straße 3, 72076 Tuebingen, Germany

**Correction to: European Radiology (2020) 30:6645–6652**

10.1007/s00330-020-07079-y

The article “Forensic age estimation in living adolescents with CT imaging of the clavicula—impact of low-dose scanning on readers’ confidence “, written by Sebastian Gassenmaier, Juergen F. Schaefer, Konstantin Nikolaou, Michael Esser and Ilias Tsiflikas, was originally published Online First without Open Access. After publication in volume 30, issue 7, page 6645–6652 the author decided to opt for Open Choice and to make the article an Open Access publication. Therefore, the copyright of the article has been changed to © The Author(s) 2020 and the article is forthwith distributed under the terms of the Creative Commons Attribution 4.0 International License, which permits use, sharing, adaptation, distribution and reproduction in any medium or format, as long as you give appropriate credit to the original author(s) and the source, provide a link to the Creative Commons license, and indicate if changes were made. The images or other third party material in this article are included in the article’s Creative Commons license, unless indicated otherwise in a credit line to the material. If material is not included in the article’s Creative Commons license and your intended use is not permitted by statutory regulation or exceeds the permitted use, you will need to obtain permission directly from the copyright holder. To view a copy of this license, visit http://creativecommons.org/licenses/by/4.0/.

